# Coronary CT Angiography Guided Medical Therapy in Subclinical Atherosclerosis

**DOI:** 10.3390/jcm10040625

**Published:** 2021-02-07

**Authors:** Alyssa L. S. Chow, Saad D. Alhassani, Andrew M. Crean, Gary R. Small

**Affiliations:** 1 Royal College of Surgeons in Ireland, Dublin 2, Ireland; AlyssaChow@rcsi.ie; 2 Department of Medicine University of Ottawa, Division of Cardiology, Ottawa, ON K1Y 4W7, Canada; salhassani@ottawaheart.ca (S.D.A.); ACrean@ottawaheart.ca (A.M.C.)

**Keywords:** primary prevention, atherosclerosis, coronary CT angiography

## Abstract

The goals of primary prevention in coronary atherosclerosis are to avoid sudden cardiac death, myocardial infarction or the need for revascularization procedures. Successful prevention will rely on accurate identification, effective therapy and monitoring of those at risk. Identification and potential monitoring can be achieved using cardiac computed tomography (CT). Cardiac CT can determine coronary artery calcification (CAC), a useful surrogate of coronary atherosclerosis burden. Cardiac CT can also assess coronary CT angiography (CCTA). CCTA can identify arterial lumen narrowing and highlight mural atherosclerosis hitherto hidden from other anatomical approaches. Herein we consider the role of CCTA and CAC-scoring in subclinical atherosclerosis. We explore the use of these modalities in screening and discuss data that has used CCTA for guiding primary prevention. We examine therapeutic trials using CCTA to determine the effects of plaque-modifying therapies. Finally, we address the role of CCTA and CAC to guide therapy as defined in current primary prevention documents. CCTA has emerged as an essential tool in the detection and management of clinical coronary artery disease. To date, its role in subclinical atherosclerosis is less well defined, yet with modern CT scanners and continued pharmacotherapy development, CCTA is likely to achieve a more prominent place in the primary prevention of coronary atherosclerosis.

## 1. Introduction

The first Chinese medical text (2600BC) foresightedly taught, “Inferior doctors treat the full-blown disease, mediocre doctors treat the disease before evident, superior doctors prevent the disease” [1]. Although as medical practitioners, we might not enjoy the implications of the statement, there is wisdom in the saying that still holds true today. In healthcare we most commonly focus on symptomatic patients, often neglecting a population who may benefit from preventative treatment. This approach, however, is changing. Increasingly, across medical specialties, we are witnessing advances in technology and imaging that present unprecedented opportunities to intervene before overt symptomatic disease occurs [2].

This is especially so in cardiovascular disease, which remains a leading cause of death worldwide, claiming 17.9 million lives per year [3] of which approximately 50% of cases are attributed to coronary heart disease [4]. To address this need, coronary CT angiography (CCTA) has gradually emerged over 20 years as an important diagnostic tool and begun to impact disease prevention [5,6,7,8]. It readily identifies both obstructive and non-obstructive coronary artery atherosclerotic plaque which is the forerunner of coronary artery disease (CAD) [9,10,11]. Not only does CCTA identify the presence of atherosclerotic plaque, it accurately risk-stratifies patients at risk of suffering coronary atherosclerotic events [12,13]. Thus, as we work to have CCTA employed in primary prevention, we strive to become the ‘superior’ doctors outlined by ancient Chinese wisdom.

In this review we consider the definition of subclinical atherosclerosis, the pathogenesis of atherosclerosis, its prevalence and current recommendations for atherosclerosis screening. We look at the application of cardiac CT in primary prevention by assessing how it: (a) refines risk prediction, (b) is employed to investigate treatment, and (c) directs therapy in primary prevention guidelines.

## 2. Subclinical versus Clinical Atherosclerosis

**Figure 1:**
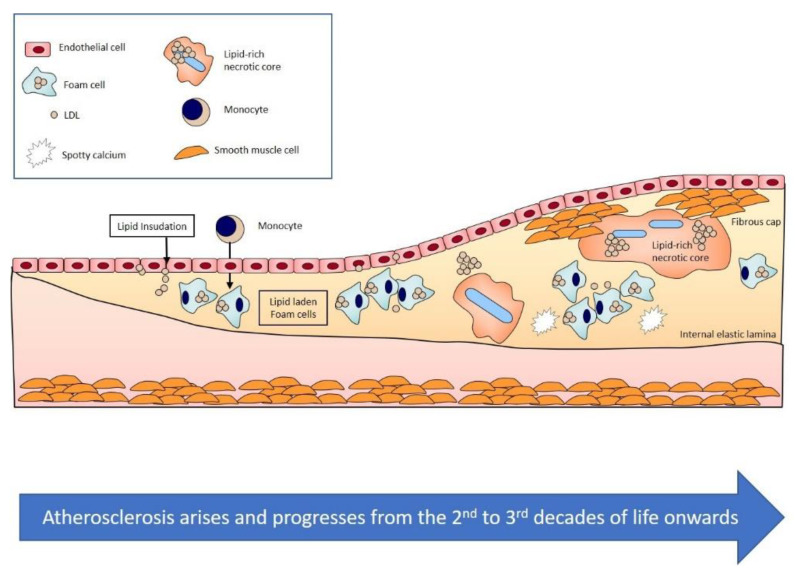
Schematic representation of atherogenesis. Atherosclerosis develops from the second and third decade of life onwards in Western populations and is characterized by the gradual accumulation of lipid-rich material within the intimal layer of arteries (Figure adapted from [14]).

Atherosclerosis is defined as an area in the intima layer of an artery that is lipid rich, contains cellular debris, lipid-laden cells, and inflammatory cells and is often calcified (Figure 1) [14]. *Clinical obstructive atherosclerosis* develops when this area encroaches upon 50% or more of the vessel lumen and causes ischemic symptoms. Alternatively, *clinical non-obstructive atherosclerosis* may also exist in the absence of a 50% luminal narrowing but following an acute plaque event that has presented as an acute coronary syndrome. In contrast, *subclinical atherosclerosis* is characterised by the presence of asymptomatic non-obstructive or obstructive atherosclerosis. 

## 3. Prevalence of Subclinical Atherosclerosis

Subclinical atherosclerosis occurs usually in the second and third decades of life in individuals from developed countries [15]. Its development is thought to reflect genetic, nutritional and cultural factors [16,17]. The disease traditionally progresses slowly for decades before the onset of symptoms. Determining prevalence of a subclinical condition can be challenging; autopsy data from American soldiers who died in combat has been instructive to assess the presence of atherosclerosis and to track the changes in the pattern of disease over time [18].

Prevalence in the young has declined over the past 60–70 years. Atherosclerosis was found in 77% of Korean war American casualties, 45% of those from the Vietnam war, and 12% in those who died in combat between 2001 and 2011 [18,19,20]. The decline speaks to the success of health behaviour and primary prevention programs [18].

The prevalence of subclinical atherosclerosis in middle and older age was studied in the Multi-Ethnic Study of Atherosclerosis (MESA) study [21]. MESA recruited 6814 participants aged 45–84 years without known cardiovascular disease and underwent coronary calcium scoring. The prevalence of subclinical atherosclerosis varied according to age, gender, and race. In non-Hispanic White Americans, the prevalence was 62%, whereas in African Americans the prevalence was 49% [22].

## 4. Primary Prevention: Screening for Atherosclerosis

The high prevalence of asymptomatic atherosclerosis emphasizes the importance of considering the condition during routine medical appointments. Guidelines recommend atherosclerosis screening for those from 40–75 years of age using clinical risk assessment algorithms [16].

## 5. Clinical Screening Tools

The Framingham Risk Score (FRS) uses traditional risk factors: hypertension, cigarette smoking, cholesterol, age and gender to predict cardiovascular risk (coronary heart disease, stroke, peripheral artery disease or heart failure) [23,24]. Originally, diabetes was included. However, in later versions diabetes was omitted since this was considered a clinical coronary heart disease risk equivalent. The FRS categorizes patients into low risk (FRS < 10%), intermediate (FRS10–20%) and high-risk (FRS > 20%) groups. The FRS 10-year cardiovascular disease risk estimate is the chosen clinical risk-stratifier in the Canadian dyslipidemia guidelines [23].

Current AHA/ACC primary prevention guidelines recommend the use of the Pooled Cohort Equations Risk Calculator (PCE) [19]. PCE predicts 10-year atherosclerotic cardiovascular disease (ASCVD) (MI and stroke both fatal and non-fatal), with an elevated risk defined as ≥7.5%. Coefficients in the PCE include those of the FRS, also incorporated are coefficients for race [25].

In Europe, the SCORE (Systematic Coronary Risk Estimation) system is recommended by the European Society of Cardiology (ESC) guidelines for dyslipidemia and cardiovascular disease prevention [5,26]. The SCORE model includes the same variables as FRS and projects a 10-year risk of fatal cardiovascular disease (fatal coronary artery disease, stroke or aneurysm) [5].

Although clinical risk scores are useful for the initial estimation of risk, they have limitations [27]. To improve risk prediction, the ESC, AHA and CCS guidelines allow for risk modifiers using cardiovascular imaging in those deemed to be at low or intermediate risk [16,23,26].

## 6. Coronary Artery Calcification and Risk Modification

Current dyslipidemia and primary prevention guidelines incorporate the use of coronary artery calcification (CAC) as a risk modifier [5,16,17,23,26] (Table 1). CAC represents mineralized atherosclerotic plaque and has been consistently demonstrated to represent the total atherosclerotic plaque burden [28,29]. Use of CAC is recommended in low-intermediate risk patients (ASCVD risk of 5–20%), particularly when the results will alter primary prevention therapy [30].

Unlike coronary calcium scoring, CCTA is not current recommended as an additional risk stratification tool with primary prevention guidelines. CCTA may detect subclinical atherosclerosis when CCTA is performed for non-coronary artery indications [31]. The utility of CCTA to help refine atherosclerosis risk was demonstrated by Pen et al. in a study that measured plaque burden using CCTA of patients with a calculated FRS. Coronary atherosclerosis was noted in 47.6% of patients in the low-risk group and in 72.7% of the intermediate risk group. In contrast, 11.7% of patients in the high FRS risk group had no visual evidence of plaque [27].

## 7. Management of Subclinical Atherosclerosis Detected by CCTA

Subclinical atherosclerotic plaque identified on CCTA may be obstructive or non-obstructive. Both are potential targets for therapy. Although obstructive lesions are often believed more likely to cause clinical events, subclinical non-obstructive plaques were responsible for subsequent myocardial infarction in 42–66% of future events in the SCOT HEART and PROMISE trials, respectively [32,33]. It would seem appropriate, therefore, whether plaques are non-obstructive or obstructive, to consider further management of subclinical atherosclerosis.

There is a paucity of randomized placebo-controlled data supporting the use of primary prevention therapies based solely on CCTA findings [34]. Rather, the clinical recommendations to prescribe primary prevention with lipid-lowering therapy is based upon corroborative findings [35,36,37,38]. The success of statins to reduce CAD events has led to the exploration of these agents in patients with cardiac CT-determined coronary atherosclerosis. The majority of this data considers the role of therapy to ameliorate the risks associated with CAC.

An investigation to demonstrate the benefit of lipid-lowering based on CAC was explored in the St Francis Heart study [39]. Arad and colleagues randomized 1005 asymptomatic individuals with CAC above the 80th percentile for age and gender to receive atorvastatin 20 mg, Vitamin C and Vitamin E daily versus matching placebos [39]. This was in addition to aspirin 81 mg oral daily. After a mean of 4.3 years of follow-up, the primary endpoint of atherosclerotic cardiovascular events was similar between the two arms of the study (6.9% versus 9.9% *p* = 0.08). In a non-prespecified subanalysis, statin therapy was associated with a reduction in cardiovascular events in those with calcium scores >400 (8.7% vs. 15% *p* = 0.046).

Henein and Owen performed a meta-analysis of 5 randomized controlled trials including a total of 1839 individuals without CAD that examined the effects of statins on CAC [34]. They also performed a meta-analysis of 6 randomized trials including a total of 1785 subjects examining the effects of statins on luminal coronary disease (LCD) (2 used intravascular ultrasound and 4 used quantitative invasive angiography). They determined that statins did not slow the progression of CAC over a period of 12–24 months [34]. Statins were however noted to reduce or slow progression of LCD. These findings have recently been confirmed in the PARADIGM study using CCTA [40].

PARADIGM investigated whether plaque volume or morphology was altered in 1255 patients with suspected or known CAD who were statin-naïve (474) or on statins (781) [40]. CCTA was performed at baseline and repeated at an interval of ≥2 years. Compared to statin-naïve patients, those on statins demonstrated a slower rate of plaque volume progression, more rapid progression of plaque calcification and developed fewer high-risk plaque features [40].

## 8. CCTA to Direct Statin Therapy in Non-Obstructive Plaque: Clinical Outcome Studies

There are two observational retrospective studies considering the clinical effects of statins in patients with subclinical atherosclerosis based upon CCTA data. Both studies used cohorts from the CONFIRM registry, whose details have been described [41]. Briefly 27,125 consecutive patients undergoing cardiac CT were enrolled in a multicentre, global registry from February 2003 to December 2009. From this registry Chow and colleagues identified 10,418 patients with either normal (5712) or non-obstructive coronary artery disease (4706) following CCTA. 33% of patients were on statins at the time of the study [10]. The presence of increasing amounts of non-obstructive plaque was associated with increasing risk for the primary endpoint of all-cause mortality. Statin use was associated with a reduced risk of mortality in those with plaque (HR 0.44 (0.28–0.68)) but not in those without (HR 0.66 (0.30–1.43)). Baseline aspirin use was not found to be beneficial at reducing the primary endpoint in this analysis [10].

Cho and colleagues also analysed the CONFIRM registry to identify those patients with non-obstructive atherosclerosis or normal coronary arteries [42]. 8016 met the inclusion criteria (there were more numerous exclusion criteria for this cohort in comparison to the study by Chow et al. [43], including the absence of a CAC score and the presence of congenital heart disease). Patients were divided into two groups depending on the use of statins at the time of the cardiac CT. CAC score was associated with an increased risk of all-cause mortality, which was attenuated by the presence of baseline statin therapy [42]. Increasing amounts of plaque as determined by CCTA using a segment involvement score was associated with increased all-cause mortality. Baseline statin use attenuated this risk [42].

Both Chow and Cho demonstrated a stepwise relationship between the amount of plaque and the degree of risk. In both circumstances, statin use attenuated these risks [10,42].

## 9. Cardiac CT to Direct Aspirin Therapy

CCTA has not shown the benefit of aspirin for primary prevention. Several studies have however suggested a promising role for CAC scoring as a decision-making tool in this regard [29,44].

The concept of using CAC to help refine the risk/benefit balance for aspirin was studied by the MESA investigators [45]. The investigators considered 4229 non-diabetic aspirin-naïve patients and determined that those with a calcium score of ≥100 had a net benefit of aspirin irrespective of their Framingham risk estimate, whereas those with a calcium score of zero had a net harm from primary aspirin prevention regardless of Framingham risk score [45].

This analysis had recently been repeated by the MESA group in response to the adoption of the PCE for clinical risk assessment of atherosclerotic cardiovascular disease (ASCVD) [46]. 3540 patients were included in the analysis, patients were excluded if they were >70 years old, had a high risk of bleeding or were on aspirin at baseline. Patients with a coronary calcium score ≥100 had net benefit of aspirin independent of the calculated ASCVD risk. In those patients with a CAC of zero there was a net harm of bleeding across the ASCVD strata [46]. CAC imaging therefore can be useful in refining the risk/benefit for primary prevention with aspirin. 

## 10. Calcium Score Zero But CCTA with Soft Plaque

**Figure 2:**
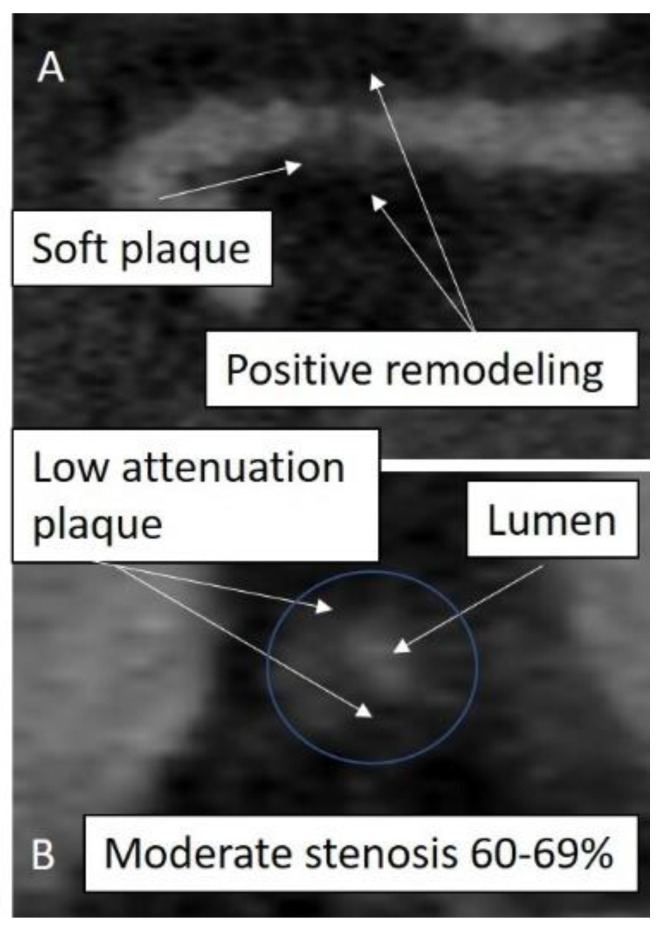
Subclinical atherosclerosis. Coronary CT angiogram (CCTA) of a patient undergoing cardiac CT prior to pulmonary vein ablation therapy. CAC was zero. A subclinical 50% stenosis was detected in the proximal left anterior descending artery (LAD) with features suggestive of an unstable plaque. Multiplanar reformatted images are shown in long axis (panel (**A**)) and short axis (panel (**B**)).

CCTA can detect ‘soft’ non-calcified plaque in patients who have a zero-calcium score (Figure 2). Management of such plaque is missing in the current AHA/ACC joint guidelines for primary prevention or dyslipidemia, although they are addressed in the ESC guidelines [16,26] (Table 1). Management is likely therefore to be determined by clinical risk stratification according to ASCVD criteria.

Studies in this cohort of patients have suggested that asymptomatic patients with non-calcific plaques and zero calcium score are usually at a low risk of subsequent cardiovascular events in the medium term. Uretsky et al. determined soft plaque to be present in 147 patients with suspected CAD in a sample of 1119 CCTA studies with a zero-calcium score [47]. The majority of patients had non-obstructive plaque; 20 patients had ≥50% stenosis. 53% of patients had a history of hypercholesterolemia. Retrospective analysis determined total mortality in the sample was 0.4% over a mean follow up of 2.5 years and did not occur in those patients with soft plaque [47].

Similar low event rates were observed more recently in 1451 low-to-intermediate ASCVD patients with suspected CAD who had undergone CCTA and had a calcium score <1 [48]. Approximately 6% of patients had soft plaques of ≥50% luminal stenosis. In this study the prevalence of high-risk plaque features was also assessed and found to be present in 8%. Mean follow up was 6.6 years and all-cause mortality was low (2.7% in patients with CAC = 0). Cardiovascular mortality was very low at 0.06%. 

Although these findings are reassuring, there are important considerations regarding the potential significance of primary prevention medications at the time of the CCTA and the presence or absence of symptoms [10,49]. Primary prevention may have attenuated subsequent clinical risk whereas the presence of anginal symptoms may have led to an increase in events. Prior investigators have indicated that symptoms are an important predictor of obstructive soft plaque in patients with zero calcium scores [49]. Although the likelihood of obstructive coronary artery disease is low when CAC is zero, it is not negligible, and further testing is often required [50,51].

## 11. Targeting Plaque Morphology with Non-Statin Therapies

Some individuals are intolerant of statin therapies or are unable to achieve primary prevention targets for LDL cholesterol reduction. In these circumstances, proprotein convertase subtilism/kexin type 9 (PCSK9) inhibitors can be useful therapeutic agents. PCSK9 inhibitors increase the activity of the LDL receptor and thereby reduce plasma LDL levels [52].

Ikegami et al. considered the effects of PCSK9 inhibitors on CAC scores in 120 patients, 60% of whom had a past history of CAD, that were divided into three groups (no therapy, statin therapy alone and dual therapy with a statin and PCSK9 inhibitor) [53]. Patients underwent calcium scoring at baseline and after one year. They determined that the median calcium score increased to the greatest degree in patients receiving statins, followed by statin-PCSK9 group and with those on no therapy, having the lowest increase in CAC. Although this was a small study the authors suggest that their results indicate that PCSK9 combined therapy may reduce the rate of coronary calcification experienced by patients on statin therapy alone [53]. Further studies will be needed to confirm this potential effect of PCSK9 inhibitors to repress statin induced coronary calcification.

## 12. Manipulating Triglycerides: Effects on Atherosclerotic Plaque Morphology

In contrast to statins and PCSK9 inhibitors that target LDL-cholesterol reduction, agents that target triglycerides, such as fibrates, have lacked consistent data for their efficacy to reduce atherosclerotic events [54,55,56,57,58]. A resurgence in interest in triglycerides has occurred following recent data that suggests omega-3 fatty acids reduce both triglyceride levels and cardiovascular events [59].

Icosapent ethyl (IPE) (a derivative of eicosapentaenoic acid, EPA), was the active therapy used in the REDUCE-IT trial. REDUCE-IT enrolled 8179 patients with either a history of CAD or were at high risk of CAD for 4.9 years and demonstrated that in comparison to placebo, in patients already treated with a statin, IPE reduced ischemic endpoints from 22% in the placebo group to 17.2% in the treatment group [59]. 

Budoff and colleagues considered the mechanisms for IPE effects by examining the influence of IPE on plaque morphology in 80 individuals noted to have coronary atherosclerosis on CCTA, already taking statins and randomly assigned to placebo versus IPE [60]. Based on CCTA assessment, IPE reduced plaque volume by 17%, and altered plaque morphology. Low attenuation plaque (−50–50 HU) volume reduced following IPE as did fibrofatty plaque (51–130 HU) and fibrous plaque (131–350 HU); there was no significant reduction in these components with placebo. There was no difference in the volume of dense calcified plaque (HU > 350) for either arm of the study [60]. 

**Figure 3:**
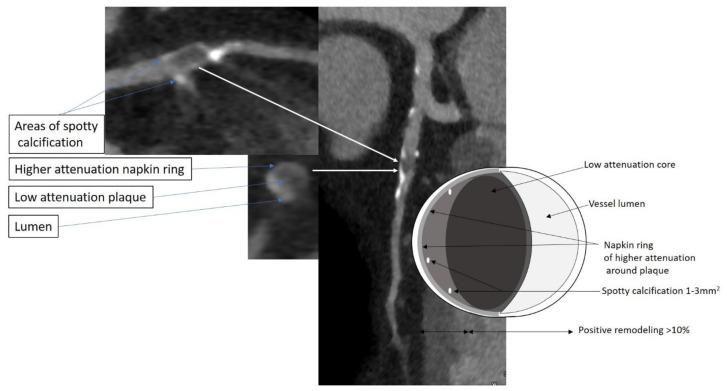
High risk plaque features. A patient with atypical chest pains underwent a CCTA. Multiplanar reformat images of the LAD are shown. An atherosclerotic plaque is seen in the proximal to mid LAD that exhibits 3 of the described hallmarks for unstable plaque: low attenuation core, a napkin ring sign and spotty calcification. A schematic cartoon demonstrating these features is illustrated.

**Figure 4:**
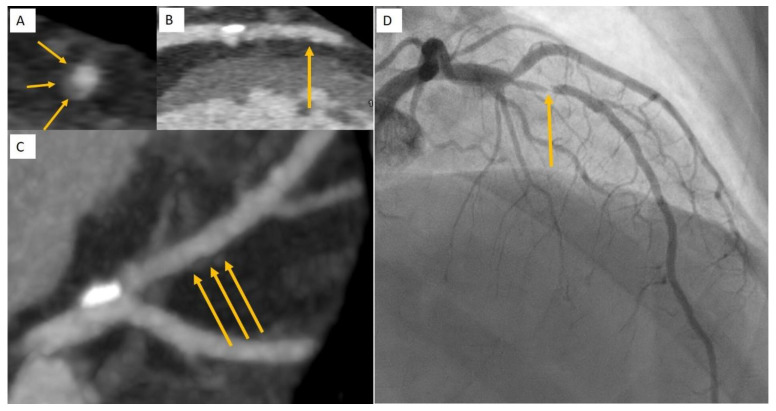
Subclinical atherosclerosis to clinical atherosclerosis. Subclinical atherosclerosis was detected during a CCTA to investigate new onset of reduced left ventricular ejection fraction of 41%. A diagnosis of non-ischemic cardiomyopathy was determined following CCTA and cardiac MRI. A mild lesion in the mid LAD was noted at CCTA to have positive remodelling (**A**), low attenuation plaque and spotty calcification (**B**,**C**) on multiplanar reformat (**A**,**B**) and maximum intensity projection (**C**). One month later the patient presented with a non-ST-elevation myocardial infarction due to an acute plaque event of the mid LAD plaque (**D**).

It has been postulated that the ability of IPE to lower ischemic events in REDUCE-IT may be due to a decrease in low attenuation plaque noted in EVAPORATE [60]. Low attenuation plaque is a robust predictor of future myocardial infarction [33,61]. Patients with low attenuation plaque burden of >4% were noted to be 4.6 times more likely to experience a myocardial infarction in a 5-year prospective CCTA study [62]. Thus, alongside statins to reduce plaque volume, IPE may add to a reduction in plaque volume and attenuate the risk of ischemic plaque events by altering plaque morphology to a more quiescent phenotype (Figure 3 and Figure 4).

## 13. Current Guidelines for Using CCTA Data in Primary Prevention

AHA/ACC guideline documents for primary prevention and dyslipidemia have been updated recently. Both are similar in terms of the recommended application of cardiac CT in managing patients with primary prevention. Although CAC can be considered in patients of low to intermediate clinical risk, there is no provision for the use of CCTA in primary prevention [16,17].

If the CAC score is 1–99, statin therapy is “favoured” for primary prevention. The risk reclassification is modest in low-intermediate risk patients with low CAC scores and the evidence for pharmacological intervention is not as strong as those with greater CAC scores. If CAC is >100, statins are recommended and in those with CAC ≥75th percentile for age, gender and race [17].

The ACC/AHA primary prevention guideline recommends low-dose aspirin use (75–100 mg) for primary prevention among adults 40–70 years of age who are at higher ASCVD risk but not at increased risk of bleeding [16,63]. The recently published MESA trial data may help to clarify for whom the risk/benefit equation is more favourable in terms of disease prevention [46].

## 14. ESC/EAA Dyslipidemia and Primary Prevention Guidelines

The European Society of Cardiology/European Atherosclerosis Association published guidelines for the management of dyslipidemia in 2019 [5]. 10-year risk estimates for fatal CVD are obtained from the SCORE charts and further refined by enhanced risk factors. Very high risk equates to a SCORE ≥10% for fatal CVD risk. Very high risk may be described on clinical grounds using the SCORE algorithm or through imaging findings such as multivessel coronary artery disease with two major coronaries having stenoses ≥50%. 

Subsequent drug intervention is recommended based on LDL lipid levels (≥1.8 mmol) in the very high-risk population, to a reduction of ≥50% from baseline and a goal of LDL < 1.4 mmol/L [5]. Reclassification of people determined to be at moderate risk by using CAC > 100 Agatston units may be considered in individuals at low or moderate risk in those patients whereby the respective LDL cholesterol goal is not achieved with lifestyle modification alone and pharmacological therapy is being considered [5]. The ESC primary prevention guidelines do not recommend antiplatelets in individuals without CVD due to the increased risk of major bleeding; further refinement of risk/benefit in individuals by calcium scoring is not discussed [26].

## 15. Canadian Dyslipidemia Guidelines

Provision for CAC scoring is made in the guidelines for intermediate risk subjects. A CAC value of 0 was considered to sufficient to reclassify such subjects as low risk, scores of >0 were determined to be associated with increasing risk of CVD. On the basis that a CAC > 100 is associated with an annual risk of >2% such individuals are classified as high risk and recommendations to intensify lipid-lowering therapy are made [23].

## 16. Conclusions

Unlike CAC, the use of CCTA for guiding primary prevention has not been widely included by guidelines. The latest European dyslipidemia guidelines have, however, adopted the use of CCTA findings and it is likely that other societies will follow suit. Data presented on the efficacy of statins, PCSK9 inhibitors and IPE to modify plaque risk markers may encourage the adoption of CCTA [64,65].

Wilson and Junger in 1968 noted at time of medical technology advancement that “screening is an admirable method of combating disease…. in practice, there are snags” [66,67]. They highlight that management of those without overt disease may seem easy however it does come with risk of inducing iatrogenic effects in those without symptoms. In attempting to resolve the potential dilemmas associated with screening Wilson and Junger formulated 10 criteria for screening (Table 2). Although the majority of these have been satisfied with regard to CAD for clinical screening, some remain unresolved for the use of CCTA or CAC in this context (for example criteria 6–10). Appropriately CAD screening with CCTA is not universally recommended at the current time.

One concern regarding CCTA screening has been the potential risks from radiation exposure [68]. Advances have reduced radiation exposures to <5 mSv for most CCTA procedures, and many CT scanners now able to image with <1 mSv. These values approach the range of mammography (0.7–1.76 mSv) [69]. Although we may not be at a stage of recommending universal screening with CCTA or CAC, it is a future possibility. With more effective plaque-modifying therapy and the use of CCTA to prevent atherosclerotic coronary artery disease, we may finally become, as the Chinese text stated, ‘superior’ doctors.

## Figures and Tables

**Table 1:** European and American Societal guidelines for use of coronary artery calcium imaging (CAC) in primary prevention. European guidance is based on the SCORE clinical algorithm that predicts 10-years risk of fatal cardiovascular (CV) disease events. Whereas the American Heart Association/American College of Cardiology (AHA/ACC) guidelines use the Pooled Cohort Equations (PCE) 10 risk of a clinical CV event.

Guideline	When to Perform Coronary Calcium Scoring	Risk	CAC Score/CT Findings and Management
2019 European Dyslipidemia Guidelines	CAC as risk modifier for asymptomatic at low or moderate risk	SCORE 10-year risk of fatal CV event Low <1% Moderate ≥1% <5%	If CAC >100 Agatston units reclassify to higher risk category: LDL target changes If >50% luminal stenosis reclassify to very-high risk category: LDL target changes
2016 European CVD Primary Prevention Guidelines	CAC as risk modifier for asymptomatic at low or moderate risk	SCORE 10-year risk of fatal CV event Low <1% Moderate ≥1% <5%	CAC ≥300 Agatston units or ≥75 percentile indicates increased risk Aspirin is not recommended
2019 ACC/AHA Guideline for Primary Prevention	Consider CAC in intermediate risk patients	PCE 10-year risk of CVD event Intermediate ≥7.5% <20%	If CAC = 0 unless enhancer present: no statin CAC = 1–99 favours statin especially if age > 55 CAC ≥ 100 and/or ≥75 percentile: statin
2018 ACC/AHA Cholesterol Clinical Practice Guidelines	Consider CAC in intermediate risk patients	PCE 10-year risk of CVD event Intermediate ≥7.5% <20%	If CAC = 0 unless enhancer present: no statin CAC = 1–99 favours statin especially if age > 55 CAC ≥ 100 and/or ≥75 percentile: statin

**Table 2:** Wilson and Junger Principles of Screening Criteria as applied to coronary artery disease (CAD).

	Principles of Early Disease Detection	CAD
1	The condition sought should be an important health problem	Satisfied
2	There should be an acceptable treatment for patients with recognized disease	Satisfied
3	Facilities for diagnosis and treatment should be available	Satisfied
4	There should be a recognizable latent or early symptomatic stage	Satisfied
5	There should be a suitable test or examination	Satisfied
6	The test should be acceptable to the population	Satisfied? Radiation concerns for CT
7	The natural history of the condition, including development from latent to declared disease, should be adequately understood	Plaque rupture events not well defined
8	There should be an agreed policy on whom to treat as patients	Some discrepancies in guidelines
9	The cost of case finding (including diagnosis and treatment of patients diagnosed) should be economically balanced in relation to possible expenditure on medical care as a whole	This is conjectural as cost of statins is low but other therapies more expensive
10	Case-finding should be a continuing process and not a once and for all project.	To screen using clinical tools widely accepted not so with CCTA

CAD, coronary artery disease.

## Data Availability

Not applicable.
